# Supplementary material for: Integration of miRNA:mRNA Co-Expression Revealed Crucial Mechanisms Modulated in Immunogenic Cancer Cell Death
Source: Biomedicines. 2022 Aug 5;10(8):1896. doi: 10.3390/biomedicines10081896 (PMC9405340; doi:10.3390/biomedicines10081896)
Supplement: Supplementary file 1 [file biomedicines-10-01896-s001.zip › biomedicines-1774098-supplementary.pdf]

Supplementary Materials

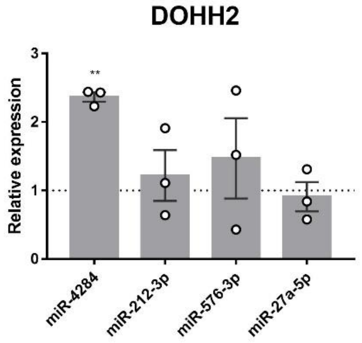

Figure S1. miRNA modulation during Immunogenic Cell Death in DOHH2 cells. DOHH2 lymphoma cells were treated with 9-cis Retinoic Acid and Interferon-alpha (RA/IFNα) for 48 hours. RT-qPCR based quantification of miRNAs expression was performed. Expression values were normalized to U6 snRNA level and referred to control untreated samples (dotted line = 1). Statistical analysis (treated vs untreated cells): One-way ANOVA, Dunnett's multiple comparisons test, ** p <0.01. Note: miR-7705 was not detected in this cell line.

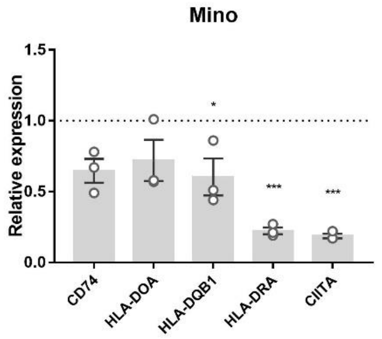

Figure S2. Modulation of Class II MHC -associated transcripts by Doxorubicin (DXR). Class II MHC-associated transcripts were evaluated by RT-qPCR in control (untreated) and DXR (ICD inducer)-treated Mino cell lines. Expression values were normalized to GAPDH levels and referred to control untreated samples (dotted line = 1). Statistical analysis (treated vs untreated cells): one-way ANOVA, Dunnett's multiple comparisons test, * p <0.05; *** p <0.001.

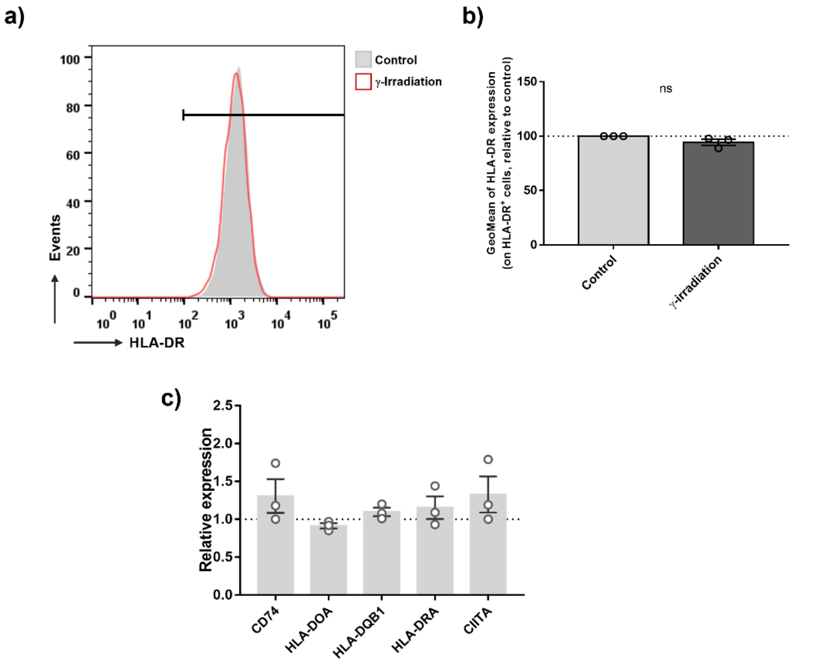

Figure S3. S. Modulation of Class II MHC pathway by gamma-irradiation (γ-irradiation). a) HLA-DR (MHC-II) surface expression was validated by flow cytometry in control (■ untreated) and γ-irradiated (□ non-ICD inducers) Mino cells. Representative histograms are shown. **b**) HLA-DR surface expression values (GeoMean) were normalized to control untreated samples (dotted line = 1). Statistical analysis (treated vs untreated cells): Student *t*-test, ns = not-significant. **c**) Class II MHC -associated transcripts were evaluated by RT-qPCR in control (untreated) γ-irradiated (□ non-ICD inducers) Mino cells. Expression values were normalized to GAPDH level and referred to control untreated samples (dotted line = 1). Statistical analysis (treated vs untreated cells): one-way ANOVA, Dunnett's multiple comparisons test (not-significant differences were observed). .
